# Data of atrial arrhythmias in hospitalized COVID-19 and influenza patients

**DOI:** 10.1016/j.dib.2022.108177

**Published:** 2022-04-14

**Authors:** Qasim Jehangir, Yi Lee, Katie Latack, Laila Poisson, Dee Dee Wang, Shiyi Song, Dinesh R. Apala, Kiritkumar Patel, Abdul R. Halabi, Geetha Krishnamoorthy, Anupam A. Sule

**Affiliations:** a Department of Medicine, St. Joseph Mercy Oakland Hospital, Pontiac, MI, United States; b Department of Public Health Sciences, Henry Ford Hospital, Detroit, MI, United States; c Division of Cardiology, Center for Structural Heart Disease, Henry Ford Hospital, Detroit, MI, United States; d Division of Cardiology, St. Joseph Mercy Oakland Hospital, Pontiac, MI, United States; e Department of Informatics, St. Joseph Mercy Oakland Hospital, Pontiac, MI, United States

**Keywords:** Atrial arrhythmias, Heart failure, COVID-19, Influenza, Echocardiography, Chest computerized tomography

## Abstract

Atrial arrhythmias (AA) are common in hospitalized COVID-19 patients with limited data on their association with COVID-19 infection, clinical and imaging outcomes. In the related research article using retrospective research data from one quaternary care and five community hospitals, patients aged 18 years and above with positive SARS-CoV-2 polymerase chain reaction test were included. 6927 patients met the inclusion criteria. The data in this article provides demographics, home medications, in-hospital events and COVID-19 treatments, multivariable generalized linear regression regression models using a log link with a Poisson distribution (multi-parameter regression [MPR]) to determine predictors of new-onset AA and mortality in COVID-19 patients, computerized tomography chest scan findings, echocardiographic findings, and International Classification of Diseases–Tenth Revision codes. The clinical outcomes were compared to a propensity-matched cohort of influenza patients. For influenza, data is reported on baseline demographics, comorbid conditions, and in-hospital events. Generalized linear regression models were built for COVID-19 patients using demographic characteristics, comorbid conditions, and presenting labs which were significantly different between the groups, and hypoxia in the emergency room. Statistical analysis was performed using R programming language (version 4, ggplot2 package). Multivariable generalized linear regression model showed that, relative to normal sinus rhythm, history of AA (adjusted relative risk [RR]: 1.38; 95% CI: 1.11–1.71; *p* = 0.003) and newly-detected AA (adjusted RR: 2.02 95% CI: 1.68–2.43; *p* < 0.001) were independently associated with higher in-hospital mortality. Age in increments of 10 years, male sex, White race, prior history of coronary artery disease, congestive heart failure, end-stage renal disease, presenting leukocytosis, hypermagnesemia, and hypomagnesemia were found to be independent predictors of new-onset AA in the MPR model.

The dataset reported is related to the research article entitled “Incidence, Mortality, and Imaging Outcomes of Atrial Arrhythmias in COVID-19” [Jehangir et al. Incidence, Mortality, and Imaging Outcomes of Atrial Arrhythmias in COVID-19, American Journal of Cardiology] [1].

## Specifications Table

**Table:** 

Subject	Cardiology and Cardiovascular Medicine
Specific subject area	Cardiac Electrophysiology
Type of data	Table Figure Supplementary datasheet
How the data were acquired	Retrospective chart and data review of hospitalized COVID-19 patients and influenza patients meeting the inclusion criteria.
Data format	Raw Analyzed
Description of data collection	Data were collected for COVID-19 and influenza patients fulfilling the inclusion criteria. COVID-19 patient records were retrospectively examined. Data pertaining to vital signs, laboratory values, baseline demographics, comorbid conditions, in-hospital COVID-19 treatments, and in-hospital events were electronically extracted from the electronic medical record. Social history, pre-admission medications, chest computed tomography findings, and echocardiographic findings were extracted manually from the electronic medical record for a subset of cases. Likewise, influenza patents records were examined and data were electronically extracted from the EMR on baseline demographics, comorbid conditions, and in-hospital events.
Data source location	Henry Ford Health System and Trinity Health System, Michigan, United States of America
Data accessibility	Supplementary file is deposited in Mendeley. Repository name: Mendeley Data identification number: data: 10.17632/rm6rjpft8j.5 Direct URL to data: https://data.mendeley.com/datasets/rm6rjpft8j/5
	Instructions for accessing these data: Retrospective data were obtained from the Epic electronic medical record covering the time period between January 1, 2014 through December 31, 2019 for patients hospitalized with influenza virus infection, and from March 1, 2020 through March 31, 2021 for patients hospitalized with SARS-CoV-2 virus infection. Patients were divided into 3 groups based on the history of atrial arrhythmias (atrial fibrillation and atrial flutter). A broad range of variables were collected for COVID-19 and influenza patients. The study had approval as a retrospective study from Henry Ford Health System and Trinity Health institutional review boards. Statistical analysis was performed using R version 4.0.4.
Related research article	Q. Jehangir, Y. Lee, K. Latack et al. Incidence, Mortality, and Imaging Outcomes of Atrial Arrhythmias in COVID-19, Am J Card. DOI: http://dx.doi.org/10.1016/j.amjcard.2022.02.051

## Value of the Data

• These data are useful as they provide insights into large and diverse populations of COVID-19 and influenza patients with atrial arrhythmias (atrial fibrillation and atrial flutter) admitted at six hospitals in Southeast Michigan.
• Our data identify risk factors of new-onset atrial arrhythmias and mortality in SARS-CoV-2 infection. In addition, we report crucial clinical and imaging findings and in-hospital treatments of COVID-19 patients.
• The data is of value to clinicians as there is limited published data on the impact of atrial arrhythmias on chest computerized tomographic and echocardiographic findings in COVID-19 infection. Moreover, literature comparing atrial arrhythmias in COVID-19 to influenza patients is deficient.
• Our data present a broad range of outcomes, including mortality, new-onset heart failure, and myocardial infarction in atrial arrhythmia patients with COVID-19, and compare the results to a propensity-matched cohort of influenza patients. These data suggest that COVID-19 is associated with a higher risk of new-onset atrial arrhythmias than influenza.
• These data have important clinical implications as new-onset atrial arrhythmias confer an unfavorable prognosis in viral pneumonia, with mortality higher in influenza than COVID-19 infection. Cardiologists, infectious disease specialists, and internists may find this data useful as early identification and treatment of atrial arrhythmias can potentially improve outcomes in viral pneumonia.
• Researchers can use data from our multicenter registry to further investigate COVID-19 and influenza patients, compare our results with other studies and perform systematic reviews and meta-analyses.

## 1 Data Description

In this study, we collected data of patients who were hospitalized with SARS-CoV-2 and influenza virus infections. The prevalance of atrial arrhythmias (AA) in COVID-19 is reported from 15.8 to 19.6% across academic centers in the United States [2], [3], [4], [5]. In COVID-19 hospitalized patients, AA are independently associated with higher in-hospital mortality [3,5]. Moreover, respiratory viruses such as influenza, severe acute respiratory syndrome coronavirus, and SARS-CoV-2 can be associated with decompensated congestive heart failure (CHF) [6], [7], [8], [9], [10]. The incidence of AA, mortality, and clinical outcomes associated with AA, including new-onset CHF, were compared between COVID-19 and influenza populations after propensity matching. Moreover, the imaging outcomes, including computerized tomography chest scan findings and transthoracic echocardiographic findings, were studied in COVID-19 patients.

**Supplementary File:** Analyzed data of COVID-19 and influenza patients admitted to the hospitals in Southeast Michigan. Patients were stratified into normal sinus rhythm (NSR), new-onset AA, and history of AA. Detailed data on baseline demographics, comorbid conditions, and in-hospital events are reported for COVID-19 and influenza cohorts. Moreover, data on vital signs, laboratory values, social history, pre-admission medications and in-hospital medical treatments, computerized chest tomography (CT), and echocardiographic data are reported for COVID-19 patients. The file can be found on Mendeley.

**Fig. 1:**
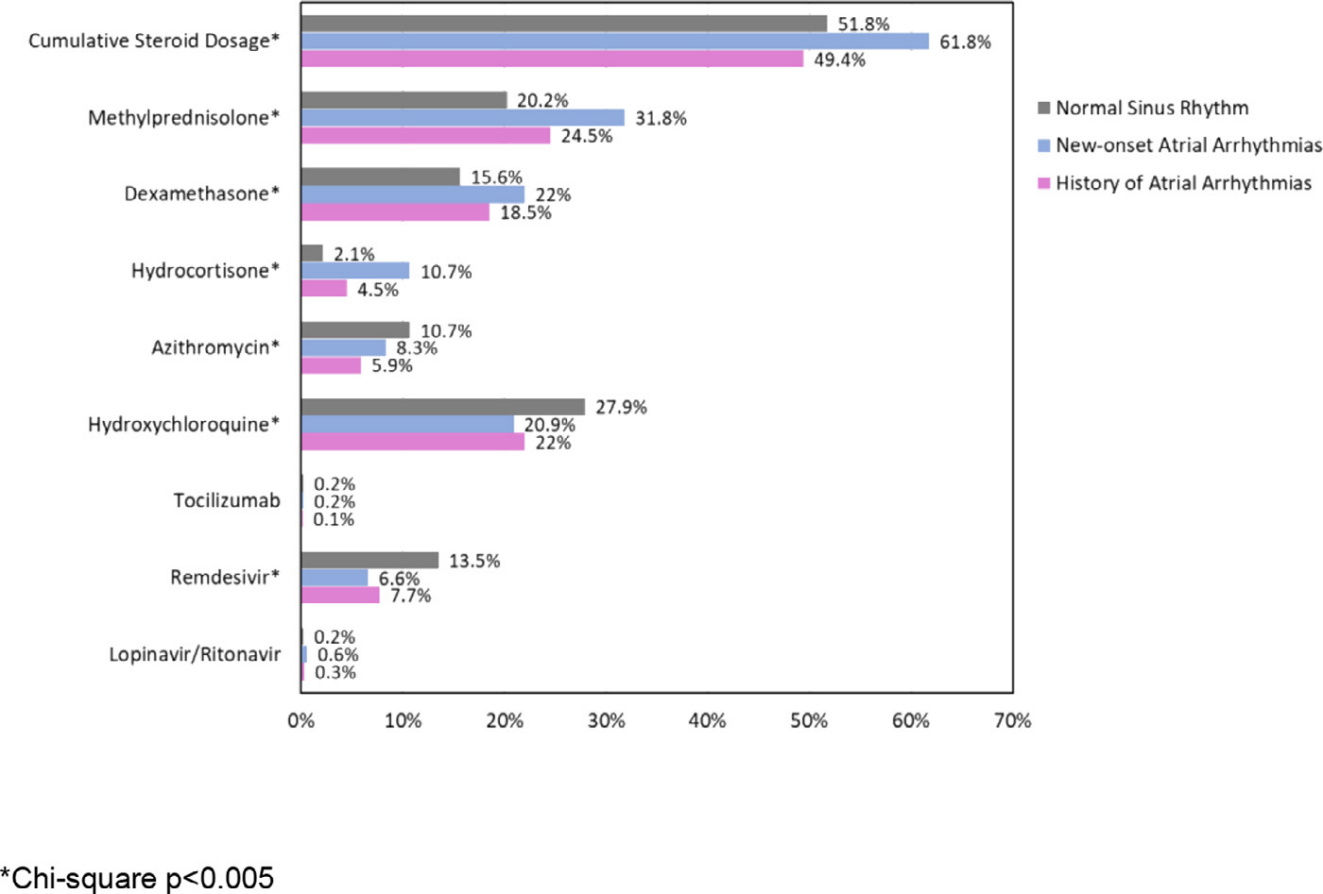
In-hospital steroids and other COVID-19 specific treatment in three groups *Chi-square *p <* 0.005.

Fig. 1 – Figure showing COVID-19 treatments received during hospitalization including lopinavir, ritonavir, remdesivir, tocilizumab, hydroxychloroquine, azithromycin, and cumulative dosage of steroids including hydrocortisone, dexamethasone, and methylprednisolone. COVID-19 patients were stratified into three groups: NSR, new-onset AA, and AA. Remdesivir and hydroxychloroquine usage were more common in NSR compared to patients with history of AA and new-onset AA. Cumulative dosage of steroids was defined as methylprednisolone 40 mg twice daily or ≥80 mg daily for ≥3 or more days; dexamethasone ≥6 mg daily for ≥3 days, and hydrocortisone ≥50 mg daily for ≥3 days. If a patient on the aforementioned dosage of steroids died before reaching 3 days length of stay, they were included in the steroids regimen group as they likely had severe COVID-19 disease. Patients on the same dosage who were discharged before reaching 3 days length of stay likely did not have severe disease and were excluded from the steroid group. The use of all steroids including hydrocortisone, dexamethasone, and methylprednisolone was more frequent in patients with new-onset AA. The difference in the usage of lopinavir, ritonavir, and tocilizumab did not reach statistical significance.

**Fig. 2:**
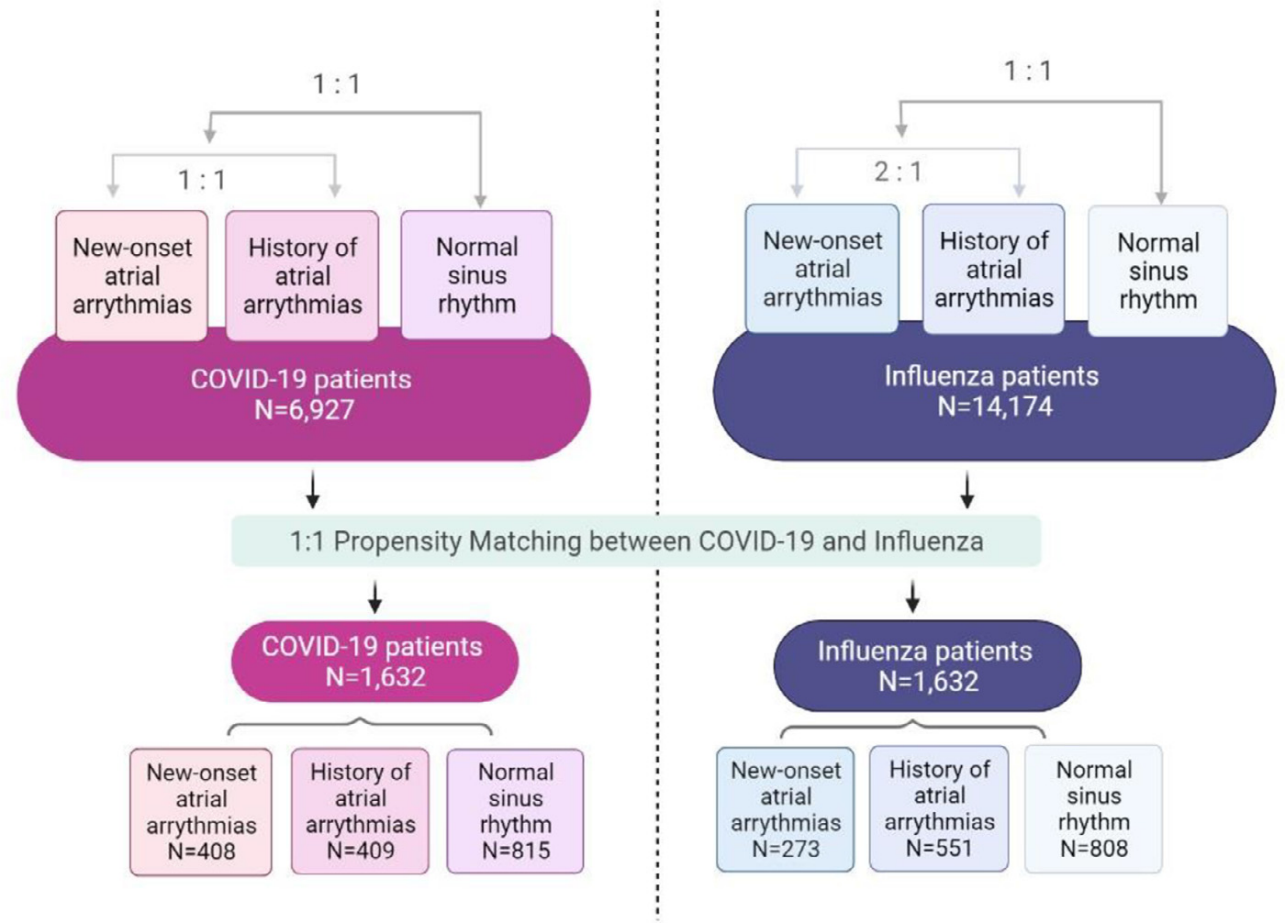
Flow diagram illustrating propensity matching between COVID-19 and influenza populations.

Fig. 2 – Figure showing the propensity matching done between COVID-19 and influenza cohorts. A total of 6,927 COVID-19 and 14,174 patients were initially included. The patients were stratified into three groups in each cohort based on the status of AA. Matches were made within population, first between new-onset and history of AA groups, then between AA (new+history) and NSR groups. Finally matches were made between COVID-19 and influenza populations. After completing the propensity matching, the cohorts had 1632 patients each.

Table 1 – Table showing International Classification of Diseases–Tenth Revision (ICD-10) codes and other identification methods used in the study. The ICD-10 codes were used to identify the patients with influenza, AA, chronic heart failure (HF), and the outcomes including new-onset HF, ischemic stroke, transient ischemic stroke, myocardial infarction (MI), deep vein thrombosis (DVT), other arterial thromboembolism, pulmonary embolism, ventricular tachycardia (VT) and ventricular fibrillation (VF), acute renal failure (ARF), requirement for new renal replacement therapy (RRT), and minor and major bleeding (using International Society on Thrombosis and Haemostasis definition) [11]. Standardized text variables were also used to identify patients with AA, chronic and new-onset HF, requirement for RRT, along with transfusion and drop in hemoglobin ≥2 mg/dL.

**Table 1:** International Classification of Diseases–Tenth Revision codes and other identification methods used for study.

Variable	ICD-10 Codes & Identification Methods
COVID-19	Variable codes from consortium database
Influenza	J09, J10, J11
Paroxysmal atrial fibrillation	I48.0
Persistent atrial fibrillation	I48.1
Chronic atrial fibrillation	I48.2
Typical atrial flutter	I48.3
Atypical atrial flutter	I48.4
Unspecified atrial fibrillation	I48.91
Unspecified atrial flutter	I48.92
New-onset heart failure	No history of heart failure in past medical history and heart failure in discharge diagnosis I50 I50.1 I50.2 I50.20 I50.21 I50.3 I50.30 I50.31 I50.4 I50.40 I50.41 I50.8 I50.81 I50.810 I50.811 I50.814 I50.82 I50.83 I50.84 I50.89 I50.9 I11.0 I13.0 I13.2 I25.5 I42 I43
Chronic heart failure	History of heart failure in past medical history I50.22 I50.23 I50.32 I50.33 I50.42 I50.43 I50.812 I50.813
Ischemic stroke	I63
Transient ischemic attack	G45 I65 I66
ST-segment elevation myocardial infarction	I21.0 I21.01 I21.02 I21.09 I21.1 I21.11 I21.19 I21.2 I21.21 I21.29 I21.3 I22.0 I22.1 I22.8 I22.9
Non-ST-segment elevation myocardial infarction	I21 I21.4 I21.9 I21.A I21.A1 I21.A9 I22.2
Deep vein thrombosis	I82 I82.211 I82.221 I82.291 I82.5 I82.7 I82.A2 I82.B2 I82.C2 I82.891 I82.91
Other arterial thromboembolism	I73.9 I74 I75
Pulmonary embolism	I26
Ventricular fibrillation	I49.0
Ventricular tachycardia	I47.2
Acute renal failure	N17 N19 R94.4
Requiring new dialysis	Variable codes from consortium database No history of end-stage renal disease and N18.6, Z99.2
Transfusion	Variable codes from consortium database
Drop in hemoglobin ≥2 mg/dL	Identified from labs
Acute posthemorrhagic anemia	D62
Hemorrhage, not elsewhere classified	R58
Hemothorax	J94.2
Intracranial hemorrhage	I60 I61 I62 I69.0 I69.1 I69.2
Intraocular bleed	H43.1 H44.81 H21.0 H35.6 H11.3 H31.30 H31.31
Hemoperitoneum/retroperitoneal bleed	K66.1
Intra-articular bleeding	M25.0
Hemopericardium	I23.0 I31.2
Intramuscular bleeding and compartment syndrome	M79.A + M79.81
Gastrointestinal bleeding	I85.01 I85.11 K22.11 K22.6 K25.0 K25.2 K25.4 K25.6 K26.0 K26.2 K26.4 K26.6 K27.0 K27.2 K27.4 K27.6 K28.0 K28.2 K28.4 K28.6 K29.01 K29.21 K29.31 K29.41 K29.51 K29.61 K29.71 K29.81 K29.91 K31.811 K55.21 K57.01 K57.11 K57.13 K57.21 K57.31 K57.33 K57.41 K57.51 K57.53 K57.81 K57.91 K57.93 K62.5 K63.81 K92.0 K92.1 K92.2
Urogenital bleeding	N02 R31.0 R31.9 N95.0 N93.9 N50.1
Respiratory passages bleeding	R04

Table 2 – Table showing the baseline demographics and comorbidities of 14,174 hospitalized influenza patients. Patients were stratified into three groups based on the status of AA. Among the influenza cohort, 12,325 remained in NSR, 1,499 patients had history of AA, whereas 350 patients experienced new-onset AA. The Kruskal-Wallis test was used for age, and Chi-square tests were used otherwise. All the baseline characteristics were significantly different between the groups (*p* < 0.001).

**Table 2:** Baseline characteristics of influenza patients.

Variable	Normal Sinus Rhythm (*N* = 12,325)	New-Onset Atrial Arrhythmias (*N =* 350)	History of Atrial Arrhythmias (*N =* 1,499)	*p*-Value
Age (years)*	38.1 (<18, 63.1)	75.6 [63.6, 85.1]	75.2 [65.6, 83.6]	**<0.001**
Women	7760 (63%)	179 (51.1%)	790 (52.7%)	**<0.001**
Men	4565 (37%)	171 (48.9%)	709 (47.3%)	**<0.001**
Black	4645 (40.5%)	113 (33.2%)	293 (19.8%)	**<0.001**
White	5989 (52.2%)	214 (62.9%)	1132 (76.6%)	
Other races	845 (7.4%)	13 (3.8%)	52 (3.5%)	
Diabetes mellitus	2634 (21.4%)	137 (39.1%)	613 (40.9%)	**<0.001**
Hypertension	5167 (41.9%)	273 (78%)	1353 (90.3%)	**<0.001**
Congestive heart failure	1165 (9.5%)	134 (38.3%)	780 (52%)	**<0.001**
Stroke/transient ischemic attack	911 (7%)	63 (18%)	383 (25%)	**<0.001**
Deep vein thrombosis	577 (4.7%)	16 (4.6%)	178 (11.9%)	**<0.001**
Pulmonary embolism	383 (3.1%)	7 (2%)	110 (7.3%)	**<0.001**
Pulmonary disease	3502 (28.4%)	119 (34%)	662 (44.2%)	**<0.001**
Chronic kidney disease	11206 (90.9%)	288 (82.3%)	1097 (73.2%)	**<0.001**
End-stage renal disease	264 (2.1%)	16 (4.6%)	70 (4.7%)	
Cancer	1566 (12.7%)	69 (19.7%)	441 (29.4%)	**<0.001**
Autoimmune disease	432 (3.5%)	18 (5.1%)	98 (6.5%)	**<0.001**
Hypothyroidism	897 (7.3%)	46 (13.1%)	309 (20.6%)	**<0.001**

⁎ Median (interquartile range).

Table 3 – Table showing the medical treatments received by COVID-19 patients with new-onset AA and history of AA during hospitalization. Chi-square test or Fisher-exact test was used based on expected cell counts. A total of 67% of patients with new-onset AA and 65% patients with history of AA received rate controlling agents during hospitalization with no statistical difference between the groups. The usage of rhythm controlling agents was more frequent in patients with new-onset AA (25%) compared to history of AA (21%).

**Table 3:** Medical treatments including rate and rhythm control therapies for atrial arrhythmias during hospitalization.

In-hospital Medications	New-onset Atrial Arrythmias (*N =* 626)	History of Atrial Arrythmias (*N =* 779)	*p*-value
Rate control agents*	420 (67%)	503 (65%)	0.32
Beta-blockers	395 (63%)	475 (61%	0.89
Esmolol	2 (0.32%)	1 (0.13%)	0.89
Calcium channel blockers	118 (19%)	111 (14%)	0.08
Digoxin	47 (8%)	57 (7%)	0.89
Rhythm control agents§	159 (25%)	160 (21%)	**0.031**
Amiodarone	147 (23%)	124 (16%)	**0.0024**
Flecainide	1 (0.16%)	10 (1.3%)	0.14
Propafenone	2 (0.3%)	1 (0.9%)	0.45
Sotalol	9 (1.4%)	20 (2.6%)	0.45
Dofetilide	1 (0.16%)	4 (0.51%)	0.45
Dronedarone	1 (0.16%)	0	0.45

⁎ Include metoprolol, carvedilol, atenolol, propranolol, nadolol, timolol and pindolol.

§ Include diltiazem and verapamil.

Table 4 – Table showing anticoagulant treatments received by COVID-19 patients during the hospitalization. Chi-square test or Fisher-exact test was used based on expected cell counts. A total of 76.6% patients with new-onset AA, 76.4% with history of AA, and 23.3% with NSR received therapeutic doses of anticoagulants. The usage of prophylactic anticoagulation was most common in patients with NSR (55.5% vs 27.7% with new-onset AA vs 15.2% with history of AA). Anticoagulation was not given in 13.1% of patients with new-onset AA, 9.2% with history of AA, and 2.8% with NSR.

**Table 4:** Anticoagulant treatment for 3 groups during hospitalization.

In-hospital Anticoagulants	Normal Sinus Rhythm (*N =* 2515)	New-onset Atrial Arrhythmias (*N =* 303)	History of Atrial Arrhythmias (*N =* 343)	*p*-value
Therapeutic anticoagulation	587 (23.3%)	232 (76.6%)	262 (76.4%)	**<0.001**
Apixaban	143 (5.7%)	105 (34.7%)	130 (37.9%)	0.052
Argatroban	9 (0.4%)	4 (1.3%)	2 (0.6%)	0.074
Bivalirudin	0	0	1 (0.3%)	0.368
Dabigatran	0	0	3 (0.9%)	0.050
Edoxaban	0	1 (33%)	0	0.368
Enoxaparin	168 (6.7%)	26 (8.6%)	28 (8.2%)	**<0.001**
Fondaparinux	9 (0.4%)	0	1 (0.3%)	**<0.001**
Heparin	384 (15.3%)	135 (44.6%)	90 (26.2%)	**<0.001**
Rivaroxaban	30 (1.2%)	13	37 (10.8%)	**0.003**
Warfarin	62 (2.5%)	28	45 (13.1%)	**0.002**
Prophylactic anticoagulation	1395 (55.5%)	36 (11.9%)	52 (15.2%)	**<0.001**
Enoxaparin	1507 (59.9%)	84 (27.7%)	76 (22.2%)	**<0.001**
Fondaparinux	1 (0.04%)	0	0	0.368
Rivaroxaban	5 (0.2%)	0	0	**0.007**
No anticoagulation	533 (2.8%)	35 (13.1%)	29 (9.2%)	**<0.001**

Table 5 – Table showing three groups within the COVID-19 population after propensity matching between the three groups. The Kruskal-Wallis test was used for age, and Chi-square tests were used otherwise. A 1:1 match (history of AA vs new-onset AA) was used, followed by a 1:1 match between AA (new-onset AA + history of AA) and NSR groups. All *p*-values are insignificant indicating successful matching.

**Table 5:** Comparability of the groups according to atrial arrhythmias status with groups matched within the COVID-19 population.

Variable	Normal Sinus Rhythm (*N =* 1,048)	New-onset Atrial Arrhythmias (*N =* 526)	History of Atrial Arrhythmias (*N =* 522)	*p*-value
Age (years)*	79 [69, 86]	77 [69, 84]	78 [70, 86]	0.270
Women	485 (46.3%)	234 (44.5%)	239 (45.8%)	0.797
Black	256 (24.4%)	126 (24%)	125 (23.9%)	0.929
White	761 (72.6%)	380 (72.2%)	379 (72.6%)	
Other races	31 (3%)	20 (3.8%)	18 (3.4%)	
Body mass index, (kg/m^2^)				0.919
<18.5	94 (9%)	43 (8.2%)	41 (7.9%)	
18.5–24.9	264 (25.2%)	122 (23.2%)	123 (23.6%)	
25.0–29.9	322 (30.7%)	165 (31.4%)	165 (31.6%)	
≥30.0	368 (35.1%)	196 (37.3%)	193 (37%)	
Diabetes mellitus	394 (37.6%)	205 (39%)	190 (36.4%)	0.690
Hypertension	858 (81.9%)	421 (80%)	422 (80.8%)	0.666
Congestive heart failure	366 (34.9%)	197 (37.5%)	202 (38.7%)	0.299
Stroke/transient ischemic attack	147 (14%)	74 (14.1%)	70 (13.4%)	0.937
Deep vein thrombosis	66 (6.3%)	34 (6.5%)	36 (6.9%)	0.902
Pulmonary embolism	29 (2.8%)	11 (2.1%)	15 (2.9%)	0.672
Pulmonary disease	313 (29.9%)	154 (29.3%)	151 (28.9%)	0.922
Chronic kidney disease	161 (15.4%)	86 (16.3%)	82 (15.7%)	0.981
End-stage renal disease	46 (4.4%)	21 (4%)	21 (4%)	
Cancer	224 (21.4%)	121 (23%)	111 (21.3%)	0.724
Autoimmune disease	43 (4.1%)	22 (4.2%)	22 (4.2%)	0.994
Hyperthyroidism	30 (2.9%)	16 (3%)	15 (2.9%)	0.979
Hypothyroidism	114 (10.9%)	60 (11.4%)	61 (11.7%)	0.880

⁎ Median (interquartile range).

Table 6 – Table showing three groups within the influenza population after propensity matching between the three groups. The Kruskal-Wallis test was used for age, and Chi-square tests were used otherwise. Since there were few new-onset AA cases in influenza population, a 2:1 match (history of AA vs new-onset AA) was used, followed by a 1:1 match between AA (new-onset AA + history of AA) and NSR. All p-values are insignificant indicating successful matching.

**Table 6:** Comparability of the groups according to atrial arrhythmias status with groups matched within influenza population.

Variable	Normal Sinus Rhythm (*N =* 936)	New-onset Atrial Arrhythmias (*N =* 313)	History of Atrial Arrhythmias (*N =* 623)	*p*-value
Age (years)*	76.0 [64.7, 85.2]	76.2 [64.5, 85.2]	75.3 [65.2, 84.5]	0.728
Women	495 (52.9%)	160 (51.1%)	338 (54.3%)	0.657
Black	276 (29.5%)	95 (30.4%)	178 (28.6%)	0.981
White	622 (66.5%)	206 (65.8%)	421 (67.6%)	
Other races	38 (4.1%)	12 (3.8%)	24 (3.9%)	
Diabetes mellitus	385 (41.1%)	123 (39.3%)	249 (40%)	0.813
Hypertension	794 (84.8%)	262 (83.7%)	514 (82.5%)	0.472
Congestive heart failure	360 (38.5%)	123 (39.3%)	253 (40.6%)	0.696
Stroke/transient ischemic attack	162 (17.3%)	59 (18.8%)	113 (18.1%)	0.804
Deep vein thrombosis	53 (5.7%)	16 (5.1%)	31 (5%)	0.824
Pulmonary embolism	20 (2.1%)	7 (2.2%)	11 (1.8%)	0.844
Pulmonary disease	374 (40%)	113 (36.1%)	245 (39.3%)	0.476
Chronic kidney disease	159 (17%)	46 (14.7%)	91 (14.6%)	0.737
End-stage renal disease	39 (4.2%)	14 (4.5%)	26 (4.2%)	
Cancer	210 (22.4%)	65 (20.8%)	130 (20.9%)	0.701
Autoimmune disease	43 (4.6%)	17 (5.4%)	33 (5.3%)	0.755
Hypothyroidism	133 (14.2%)	43 (13.7%)	82 (13.2%)	0.841

⁎ Median (interquartile range).

Table 7 – Table showing COVID-19 and influenza cohorts after propensity matching the two populations. The Kruskal-Wallis test was used for age and Chi-square tests otherwise. A 1:1 match was used between the COVID-19 and Influenza cohorts that were already balanced on AA group (Tables 5 and 6). All p-values are insignificant indicating successful matching.

**Table 7:** Comparability of the groups according to atrial arrhythmias status with groups propensity-matched between COVID-19 and Influenza populations

	COVID-19			Influenza			
Variable	Normal Sinus Rhythm (*N =* 815)	New-onset Atrial Arrhythmias (*N =* 408)	History of Atrial Arrhythmias (*N =* 409)	Normal Sinus Rhythm (*N =* 808)	New-onset Atrial Arrhythmias (*N =* 273)	History of Atrial Arrhythmias (*N =* 551)	*p*-value
Age (years)*	77 (67, 85)	76 (68, 83)	76 (68, 84)	77.5 (66.6, 86.3)	77.6 (66.0, 85.7)	76.5 (66.4, 85.3)	0.54
Female	405 (49.7%)	206 (50.5%)	212 (51.8%)	400 (49.5%)	133 (48.7%)	279 (50.6%)	0.966
Black	216 (26.5%)	116 (28.4%)	114 (27.9%)	214 (26.5%)	77 (28.2%)	136 (24.7%)	0.984
White	567 (69.6%)	276 (67.6%)	281 (68.7%)	564 (69.8%)	187 (68.5%)	392 (71.1%)	
Other races	32 (3.9%)	16 (3.9%)	14 (3.4%)	30 (3.7%)	9 (3.3%)	23 (4.2%)	
Diabetes mellitus	319 (39.1%)	167 (40.9%)	159 (38.9%)	319 (39.5%)	106 (38.8%)	215 (39%)	0.99
Hypertension	681 (83.6%)	338 (82.8%)	340 (83.1%)	681 (84.3%)	224 (82.1%)	446 (80.9%)	0.706
Congestive heart failure	302 (37.1%)	165 (40.4%)	177 (43.3%)	302 (37.4%)	98 (35.9%)	217 (39.4%)	0.246
Stroke/transient ischemic attack	126 (15.5%)	67 (16.4%)	63 (15.4%)	125 (15.5%)	41 (15%)	81 (14.7%)	0.989
Deep vein thrombosis	55 (6.7%)	26 (6.4%)	25 (6.1%)	52 (6.4%)	14 (5.1%)	29 (5.3%)	0.863
Pulmonary embolism	21 (2.6%)	5 (1.2%)	11 (2.7%)	19 (2.4%)	6 (2.2%)	11 (2%)	0.713
Pulmonary disease	285 (35%)	139 (34.1%)	145 (35.5%)	282 (34.9%)	87 (31.9%)	196 (35.6%)	0.928
Chronic kidney Disease	149 (18.3%)	68 (16.7%)	68 (16.6%)	148 (18.3%)	39 (14.3%)	84 (15.2%)	0.831
End-stage renal disease	36 (4.4%)	19 (4.7%)	16 (3.9%)	34 (4.2%)	13 (4.8%)	20 (3.6%)	
Cancer	174 (21.3%)	101 (24.8%)	87 (21.3%)	192 (23.8%)	57 (20.9%)	121 (22%)	0.638
Hypothyroidism	93 (11.4%)	56 (13.7%)	57 (13.9%)	97 (12%)	35 (12.8%)	64 (11.6%)	0.738

⁎ Median (interquartile range).

Table 8 – Table showing the pre-hospital medications in the study population. Usage of statins, warfarin, direct oral anticoagulants, digoxin, beta-blockers, and diuretics were more common in patients with history of AA whereas antiplatelets usage was more common in patients with new-onset AA. There was no difference in the use of angiotensin-converting-enzyme inhibitors, angiotensin receptor blockers, calcium channel blockers, azithromycin, and hydroxychloroquine between the study groups.

**Table 8:** Medication usage prior to admission.

Variable	Normal Sinus Rhythm (*N =* 747)	New-onset Atrial Arrythmias (*N =* 76)	History of Atrial Arrythmias (*N =* 95)	Chi-square *p*-value
Home statins	289 (38.7%)	39 (51.3%)	59 (61%)	**<0.0001**
Home ACE inhibitors and ARBs	256 (34.3%)	33 (34.3%)	39 (41%)	0.296
Home warfarin	28 (3.8%)	6 (7.9%)	13 (13.7%)	**0.0003**
Home direct oral anticoagulants	21 (2.8%)	14 (18.4%)	34 (35.8%)	**<.0001**
Home digoxin	3 (0.4%)	4 (5.3%)	6 (6.3%)	**<0.0001**
Home beta-blockers	211 (28.3%)	40 (52.6%)	56 (59%)	**<0.0001**
Home diuretics	182 (24.4%)	31 (40.8%)	45 (47.4%)	**<0.0001**
Home calcium channel blockers	9 (2.11%)	2 (1.2%)	2 (2.63%)	0.505
Home antiplatelets	217 (29%)	41 (54%)	42 (44%)	**<0.0001**
Home azithromycin	57 (7.6%)	10 (13.2%)	2 (2.1%)	0.0943
Home hydroxychloroquine	15 (2%)	3 (4%)	4 (4.2%)	0.340

Abbreviations: ACE, angiotensin-converting-enzyme inhibitor; ARB, angiotensin receptor blockers.

Table 9 – Table showing multivariable generalized linear regression model using a log link with a Poisson distribution (multi-parameter regression [MPR]) built to identify predictors of new-onset AA. The model was built using baseline demographic characteristics (age, sex, race, body mass index), comorbid conditions (hypertension, diabetes mellitus, CHF, cerebrovascular accident, kidney disease, pulmonary disease, pulmonary hypertension, liver disease, cancer, thyroid disease, and history of DVT) and on-arrival labs (white cell count, aspartate aminotransferase (AST), D-dimer, potassium, and magnesium) which were significantly different between the groups, and hypoxia in the emergency room. Adjusted relative risk (RR) with 95% confidence intervals (CI) were calculated. Significant variables included: age in increments of 10 years (RR: 1.60; 95% CI: 1.46–1.74; *p* < 0.001), female sex (RR: 0.69; 95% CI: 0.57–0.85; *p* < 0.001), African American race (RR: 0.72; 95% CI: 0.57–0.92; *p* = 0.007), other races (RR: 0.56; 95% CI: 0.34–0.94; *p* = 0.027), CHF (RR: 1.55; 95% CI: 1.17–2.06; *p* = 0.002), end-stage renal disease (ESRD) (RR: 1.93; 95% CI: 1.27 – 2.93; *p* = 0.002), presenting leukocytosis (RR: 1.49; 95% CI: 1.17–1.88; *p <* 0.001), hypermagnesemia (RR: 1.46; 95% CI: 1.01–2.13; *p* = 0.047), and hypomagnesemia (RR: 1.29; 95% CI: 1.02–1.63; *p* = 0.034).

**Table 9:** Multivariable generalized linear regression model using a log link with a Poisson distribution for predictors of new-onset atrial arrhythmias.

Covariate	Relative Risk	95%Confidence Interval	*p*-value	Type 3 *p*-value
**Age (decade)**	1.60	[1.46–1.74]	**<0.001**	**<0.001**
**Body mass index**				0.079
≥30.0	1.30	[0.98–1.73]	0.065	
25.0–29.9	1.05	[0.69–1.59]	0.824	
<18.5	1.41	[1.06–1.88]	0.020	
18.5–24.9	-	-	-	
**Gender**				
Female	0.69	[0.57–0.85]	**<0.001**	**<0.001**
Male	-	-	-	
**Race**				
African American	0.72	[0.57–0.92]	**0.007**	**0.004**
Other races	0.56	[0.34–0.94]	**0.027**	
White	-	-	-	
**Hypoxia**^c^	1.14	[0.93–1.41]	0.215	0.213
**Presenting white cell count**				
High	1.49	[1.17–1.88]	**<0.001**	**0.006**
Low	1.03	[0.72–1.48]	0.869	
**Presenting aspartate aminotransferase**				
High	1.11	[0.91–1.37]	0.305	0.580
Low	0.97	[0.51–1.84]	0.869	
**Presenting D-Dimer**^b^				
High*	0.93	[0.74–1.16]	0.510	0.511
Very high**	1.08	[0.79–1.47]	0.632	
**Presenting serum potassium**				
Hyperkalemia	1.15	[0.83–1.60]	0.408	0.647
Hypokalemia	0.95	[0.72–1.25]	0.718	
**Presenting serum magnesium**				
Hypermagnesemia	1.46	[1.01–2.13]	**0.047**	**0.026**
Hypomagnesemia	1.29	[1.02–1.63]	**0.034**	
**Comorbidities**				
Congestive heart failure	1.55	[1.17–2.06]	**0.002**	**0.003**
Diabetes mellitus	0.93	[0.76–1.16]	0.529	0.528
Chronic kidney disease	0.89	[0.66–1.20]	0.432	0.427
End-stage renal disease	1.93	[1.27–2.93]	**0.002**	**0.003**
Hypertension	1.07	[0.83–1.37]	0.615	0.614
Pulmonary disease	1.37	[1.10–1.71]	**0.005**	**0.006**
Liver disease	1.09	[0.57–2.08]	0.792	0.795
History of cancer	1.01	[0.79–1.28]	0.942	0.942
Cerebrovascular accident	0.93	[0.69–1.25]	0.615	0.612
History of deep vein thrombosis	1.35	[0.89–2.05]	0.155	0.172
Pulmonary hypertension	1.53	[0.67–3.48]	0.315	0.345
Hyperthyroidism	1.56	[0.64–3.82]	0.325	0.358
Hypothyroidism	0.11	[0.83–1.48]	0.491	0.469

Number of observations in the original data set = 6148.

Number of observations used = 4006.

Coronary artery disease (CAD) history was not included in the model due to missing values to avoid reducing the number of patients in the analysis.

⁎ High: >500–2000 ng/mL.

⁎⁎ Very high: >2000 ng/mL.

b Fibrinogen-equivalent units (FEU).

c Oxygen saturation < 95%.

Table 10 – Table showing chest CT findings in study patients. Data on pleural effusion, ground-glass infiltrates, multifocal pneumonia, pulmonary edema, and pulmonary vascular congestion was collected. Pleural effusions were most common in patients with history of AA (54.6%) compared to patients with NSR (13.8%) and new-onset AA (13.3%); *p*-value=0.02. The difference in findings of pulmonary edema, pulmonary vascular congestion, ground-glass opacities and multifocal pneumonia did not reach statistical significance.

**Table 10:** Computerized tomography chest scans during hospitalization.

Variable	Normal Sinus Rhythm (*N =* 123)	New-onset Atrial Arrythmias (*N =* 15)	History of Atrial Arrythmias (*N =* 11)	Fisher-exact *p*-value
Ground-glass infiltrates and multifocal pneumonia	60 (48.8%)	10 (66.8%)	3 (27.3%)	0.16
Pleural effusions	17 (13.8%)	2 (13.3%)	6 (54.6%)	**0.02**
Pulmonary edema and pulmonary vascular congestion	1 (0.8%)	1 (6.7%)	1 (9.1%)	0.16

Table 11 – Table showing MPR model for predictors of in-hospital mortality in COVID-19 patients. The model was built using the same variables as described in Table 9. Adjusted relative risk with 95% CI were calculated. History of AA (RR: 1.38; 95% CI 1.11–1.71; *p* = 0.003) and new-onset AA (RR: 2.02; 95% CI: 1.68–2.43; *p <* 0.001) were independent predictors of mortality. Other significant variables included: age in increments of 10 years (RR: 1.44; 95% CI: 1.34–1.54; *p <* 0.001), obesity (RR: 0.77; 95% CI: 0.62–0.95; *p* = 0.014), female sex (RR: 0.76; 95% CI: 0.65–0.88; *p <* 0.001), presenting leukocytosis (RR: 1.23; 95% CI: 1.03–1.47; *p* = 0.020), elevated AST (RR: 1.37; 95% CI: 1.18 –1.60; *p <* 0.001), high D-dimer (RR: 1.44; 95% CI: 1.19–1.74; *p <* 0.001) and very high D-dimer (RR: 1.65; 95% CI: 1.30–2.10; *p <* 0.001), hypermagnesemia (RR: 1.36; 95% CI: 1.06–1.76; *p* = 0.017), history of CHF (RR: 1.23; 95% CI: 1.00–1.51; *p* = 0.047, ESRD (RR: 1.63; 95% CI: 1.21–2.20; *p* = 0.001), cancer (RR: 1.21; 95% CI: 1.02–1.44; *p* = 0.032), and DVT (RR: 1.73; 95% CI: 1.31–2.30; *p <* 0.001).

**Table 11:** Multivariable generalized linear regression model using a log link with a Poisson distribution for predictors of in-hospital mortality in COVID-19 patients.

Covariate	Relative Risk	95% Confidence Interval	*p*-value	Type 3 p-value
**Groups based on arrythmias status**				
History of atrial arrythmias	1.38	[1.11–1.71]	**0.003**	**<0.001**
New-onset atrial arrythmias	2.02	[1.68–2.43]	**<0.001**	
Normal sinus rhythm	-	-	-	
**Age (Decade)**	1.44	[1.34–1.54]	**<0.001**	**<0.001**
**Body mass index**				
≥30.0	0.77	[0.62–0.95]	**0.014**	**0.035**
25.0–29.9	0.93	[0.76–1.13]	0.470	
<18.5	1.06	[0.81–1.39]	0.667	
18.5–24.9	-	-	-	
**Gender**				
Female	0.76	[0.65–0.88]	**<0.001**	**<0.001**
Male	-	-	-	
**Race**				
African American	0.93	[0.78–1.11]	0.431	0.381
Other races	0.79	[0.55–1.14]	0.215	
White	-	-	-	
**Presenting white cell count**				
High	1.23	[1.03–1.47]	**0.020**	0.069
Low	1.00	[0.75– 1.34]	0.986	
**Presenting aspartate aminotransferase**				
High	1.37	[1.18–1.60]	**<0.001**	**<0.001**
Low	0.75	[0.42–1.34]	0.331	
**Presenting D-Dimer**^c^				
High*	1.44	[1.19–1.74]	**<0.001**	**<0.001**
Very high⁎⁎	1.65	[1.30– 2.10]	**<0.001**	
**Presenting serum potassium**				
Hyperkalemia	1.24	[0.98–1.56]	0.069	0.097
Hypokalemia	0.89	[0.72–1.11]	0.320	
**Presenting serum magnesium**				
Hypermagnesemia	1.36	[1.06–1.76]	**0.017**	**0.023**
Hypomagnesemia	1.18	[0.98–1.42]	0.082	
**Comorbidities**				
Congestive heart failure	1.23	[1.00–1.51]	**0.047**	0.050
Diabetes mellitus	1.01	[0.86–1.19]	0.863	0.863
Chronic kidney disease	0.95	[0.77–1.17]	0.620	0.618
End-stage renal disease	1.63	[1.21–2.20]	**0.001**	**0.002**
Hypertension	1.04	[0.86–1.26]	0.705	0.705
Pulmonary disease	1.15	[0.97–1.36]	0.118	0.121
Liver disease	1.31	[0.83–2.06]	0.251	0.269
History of cancer	1.21	[1.02–1.44]	**0.032**	**0.034**
Cerebrovascular accident	1.07	[0.88–1.32]	0.491	0.494
History of deep vein thrombosis	1.73	[1.31–2.30]	**<0.001**	**<0.001**
Pulmonary hypertension	0.76	[0.38–1.55]	0.453	0.433
Hyperthyroidism	1.23	[0.58–2.61]	0.586	0.598
Hypothyroidism	0.97	[0.77–1.22]	0.779	0.778

Number of observations in the original data set = 6927.

Number of observations used = 4469.

Coronary artery disease history was not included in the model due to missing values to avoid reducing the number of patients in the analysis.

⁎ High: >500–2000 ng/mL.

⁎⁎ Very high: >2000 ng/mL.

c FEU.

Table 12 – Table showing the in-hospital events in 3 groups after propensity-matched between COVID-19 and influenza populations. The Kruskal-Wallis test was used for comparing the length of stay variables and Chi-square tests were used otherwise. A Bonferroni corrected threshold for significance within COVID-19 or influenza is 0.002 (calculated as 0.05/19). Tests attaining this threshold have p-values highlighted with bold font. Within COVID-19 population, new-onset AA patients had longer Hospital length of stay and higher incidence of intensive care unit (ICU) admission, need for mechanical ventilation, usage of vasopressors and inotropes, new-onset CHF, ARF, and VT compared to patients with history of AA and NSR. Similarly, in the influenza population, the need for mechanical ventilation, usage of vasopressors and inotropes, new-onset CHF, ST-segment elevation MI, non-ST segment elevation MI, ARF, VF, and VT were more common in patients with new-onset AA.

**Table 12:** In-hospital events in 3 groups after propensity matching on AA groups within and between COVID-19 and influenza populations.

	COVID-19				Influenza			
Variable	Normal Sinus Rhythm (*N =* 815)	New-onset Atrial Arrhythmias (*N =* 408)	History of Atrial Arrhythmias (*N =* 409)	*p*-value	Normal Sinus Rhythm (*N =* 808)	New-onset Atrial Arrhythmias (*N =* 273)	History of Atrial Arrhythmias (*N =* 551)	p-value
Hospital length of stay*	5.6 (3.4, 9.8)	8.0 (4.5, 15.1)	6.3 (4.0, 11.3)	**<0.001**	3 (2, 5)	3 (2, 6)	3 (2, 5)	0.058
Intensive care unit admission	178 (21.8%)	178 (43.6%)	103 (25.2%)	**<0.001**	33 (4.1%)	20 (7.3%)	21 (3.8%)	0.051
Intensive care unit length of stay*	5 (3, 13)	8 (3, 16)	5 (3, 10)	0.043	2 (1, 3)	3 (1, 7)	3 (1.5, 4.5)	0.117
Hospital readmission within 90 days	94 (11.5%)	32 (7.8%)	53 (13%)	0.050	321 (39.7%)	131 (48.0%)	249 (45.2%)	0.025
Respiratory failure requiring mechanical ventilation	99 (12.1%)	108 (26.5%)	52 (12.7%)	**<0.001**	10 (1.2%)	13 (4.8%)	7 (1.3%)	**<0.001**
Days on ventilator*	8 (3, 14)	9 (4.75, 16)	6.5 (3, 15)	0.181	1 (1, 2.5)	10 (1, 18)	4.5 (3.25, 5.75)	0.171
Vasopressors/inotropes usage	153 (18.8%)	146 (35.8%)	100 (24.4%)	**<0.001**	16 (2%)	14 (5.1%)	4 (0.7%)	**<0.001**
New-onset congestive heart failure	23 (2.8%)	36 (8.8%)	26 (6.4%)	**<0.001**	36 (4.5%)	38 (13.9%)	40 (7.3%)	**<0.001**
Transient ischemic attack and ischemic stroke	27 (3.3%)	14 (3.4%)	16 (3.9%)	0.862	63 (7.8%)	13 (4.8%)	35 (6.4%)	0.199
ST-segment elevation myocardial infarction	1 (0.1%)	3 (0.7%)	0	0.063	4 (0.5%)	7 (2.6%)	2 (0.4%)	**0.001**
Non-ST-segment elevation myocardial infarction	86 (10.6%)	66 (16.2%)	44 (10.8%)	0.011	71 (8.8%)	34 (12.5%)	29 (5.3%)	**0.001**
Other arterial thromboembolism	29 (3.6%)	16 (3.9%)	23 (5.6%)	0.224	45 (5.6%)	12 (4.4%)	23 (4.2%)	0.461
Deep vein thrombosis	30 (3.7%)	15 (3.7%)	6 (1.5%)	0.084	23 (2.8%)	13 (4.8%)	14 (2.5%)	0.193
Pulmonary embolism	36 (4.4%)	18 (4.4%)	10 (2.4%)	0.206	18 (2.2%)	8 (2.9%)	5 (0.9%)	0.085
Acute renal failure	334 (41%)	210 (51.5%)	169 (41.3%)	**0.001**	227 (28.1%)	94 (34.4%)	118 (21.4%)	**<0.001**
Renal failure requiring new renal replacement therapy	52 (6.4%)	38 (9.3%)	22 (5.4%)	0.063	30 (3.7%)	19 (7%)	18 (3.3%)	0.031
Ventricular fibrillation	2 (0.2%)	4 (1%)	3 (0.7%)	0.222	2 (0.2%)	7 (2.6%)	2 (0.4%)	**<0.001**
Ventricular tachycardia	23 (2.8%)	28 (6.9%)	24 (5.9%)	**0.002**	12 (1.5%)	19 (7%)	20 (3.6%)	**<0.001**

⁎ Median (interquartile range).

Table 13 – Table showing odds ratios (OR) of the in-hospitals events in 3 groups after propensity matching between COVID-19 and influenza populations. Odds ratios were calculated for each 2-group comparison using univariate logistic regression within the COVID-19 and influenza populations. Group 1 includes patients with NSR, group 2 includes patients with new-onset AA, whereas group 3 includes patients with history of AA. Within the COVID-19 population, new-onset AA had higher ICU admission rate, 90 day-readmission, need for mechanical ventilation, vasopressors and inotropes usage, new-onset CHF, non-ST-segment elevation MI, ARF, and VT as evident by OR with 95% CI not crossing 1 and *p*-value <0.05. Similar in the influenza population, new-onset AA had higher ICU admission, 90 day-readmission, need for mechanical ventilation, vasopressor and inotropes usage, new-onset CHF, ST-segment elevation MI, and requirement for new RRT, VF, and VT as shown by statistically significant OR with 95% CI.

**Table 13:** Odds ratios of the in-hospitals events in 3 groups after propensity matching on AA groups within and between COVID-19 and influenza populations.

	COVID-19			Influenza		
	Odds Ratio			Odds Ratio		
	95% Confidence Interval, *p*-value			95% Confidence Interval, *p*-value		
Variable	Group 3 vs Group 1	Group 2 vs Group 1	Group 2 vs Group 3	Group 3 vs Group 1	Group 2 vs Group 1	Group 2 vs Group 3
Intensive care unit admission	1.2 (0.9, 1.6), 0.1952	2.77 (2.12, 3.61), **<0.0001**	2.3 (1.69, 3.13), **<0.0001**	0.93 (0.51, 1.68), 0.8878	1.86 (0.99, 3.4), **0.0359**	1.99 (1.01, 3.94), **0.0398**
Readmission by 90 days	1.14 (0.78, 1.66), 0.5142	0.65 (0.41, 1.01), **0.0464**	0.57 (0.35, 0.93), **0.0215**	1.25 (1, 1.57), 0.05	1.4 (1.05, 1.86), **0.0191**	1.12 (0.83, 1.51), 0.4586
Respiratory failure requiring mechanical ventilation	1.05 (0.72, 1.53), 0.7827	2.6 (1.9, 3.57), **<0.0001**	2.47 (1.69, 3.63), **<0.0001**	1.03 (0.33, 3.01), 1	3.98 (1.59, 10.28), **0.0012**	3.88 (1.42, 11.62), **0.0034**
Vasopressors/inotropes usage	1.4 (1.04, 1.88), **0.0246**	2.41 (1.83, 3.18), **<0.0001**	1.72 (1.26, 2.36), **0.0004**	0.36 (0.09, 1.13), 0.0678	2.67 (1.19, 5.93), **0.0098**	7.37 (2.29, 31.07), **<0.0001**
New-onset congestive heart failure	2.34 (1.26, 4.35), **0.0048**	3.33 (1.89, 5.98), **<0.0001**	1.42 (0.82, 2.51), 0.1894	1.68 (1.03, 2.75), **0.0304**	3.46 (2.08, 5.76), **<0.0001**	2.06 (1.25, 3.4), **0.0034**
Transient ischemic attack and ischemic stroke	1.19 (0.59, 2.32), 0.6227	1.04 (0.5, 2.08), >0.999	0.87 (0.39, 1.94), 0.8528	0.8 (0.51, 1.25), 0.3376	0.59 (0.29, 1.11), 0.1006	0.74 (0.35, 1.46), 0.4305
ST-segment elevation myocardial infarction*	-	-	-	0.73 (0.07, 5.13), 1	5.28 (1.33, 24.79), **0.0077**	7.21 (1.36, 71.71), **0.0077**
Non-ST-segment elevation myocardial infarction	1.02 (0.68, 1.52), **0.9218**	1.64 (1.14, 2.34), **0.0058**	1.6 (1.04, 2.47), **0.0244**	0.58 (0.36, 0.92), **0.0149**	1.48 (0.93, 2.32), 0.0972	2.56 (1.47, 4.46), **<0.0001**
Deep vein thrombosis	0.39 (0.13, 0.96), **0.0313**	1 (0.49, 1.94), >0.999	2.56 (0.93, 8.14), **0.0494**	0.89 (0.42, 1.82), 0.8655	1.71 (0.78, 3.57), 0.1697	1.92 (0.82, 4.47), 0.0993
Pulmonary embolism	0.54 (0.24, 1.13), 0.1102	1 (0.53, 1.83), >0.999	1.84 (0.79, 4.52), 0.1291	0.4 (0.12, 1.13), 0.0852	1.32 (0.49, 3.25), 0.4979	3.29 (0.94, 12.91), **0.0374**
Acute renal failure	1.01 (0.79, 1.3), 0.9509	1.53 (1.19, 1.95), **0.0006**	1.51 (1.13, 2.01), **0.004**	0.7 (0.54, 0.91), **0.0063**	1.34 (0.99, 1.82), 0.0553	1.93 (1.38, 2.69), **<0.0001**
Renal failure requiring new renal replacement therapy	0.83 (0.48, 1.42), 0.5272	1.51 (0.95, 2.38), 0.0808	1.81 (1.02, 3.27), **0.0325**	0.88 (0.45, 1.64), 0.7652	1.94 (1.01, 3.63), **0.0417**	2.21 (1.08, 4.56), **0.0199**
Ventricular fibrillation	3 (0.34, 36.09), 0.3410	4.02 (0.57, 44.61), 0.0999	1.34 (0.23, 9.2), 0.7252	1.47 (0.11, 20.3), 1	10.58 (2, 105), **0.0014**	7.21 (1.36, 71.71), **0.0077**
Ventricular tachycardia	2.15 (1.14, 4.03), **0.0114**	2.54 (1.39, 4.68), **0.0013**	1.18 (0.65, 2.17), 0.5703	2.5 (1.15, 5.65), **0.0165**	4.95 (2.25, 11.36), **<0.0001**	1.98 (0.98, 3.99), **0.0377**

ST-segment elevation myocardial infarction for COVID-19 population had too few events for valid estimation of odds ratio and thus not reported.

## 2 Experimental Design, Materials and Methods

We collected data for COVID-19 and influenza patients from one quaternary care and five community hospitals at Henry Ford Health System and Trinity Health System. The first hospital admission per case was retained for both COVID-19 and influenza patients. For COVID-19 patients, clinical data were abstracted from the Epic, Inc. electronic medical record (EMR) at contributing hospitals, deidentified and stored in the Southeast Michigan COVID-19 Consortium Registry Database (SMCRD) using REDCap (software hosted at Vanderbilt University Medical Center in Nashville, Tennessee). The two systems submitted Michigan Health Information Network ID numbers (MiHIN) so that data from patients receiving care at both institutions could be linked. COVID-19 data were collected retrospectively and concurrently from 1st March 2020 to 31st March 2021. Hospitalized patients aged 18 years and above with polymerase chain reaction-proven SARS-CoV-2 infection were included. Out of 6943 patients in the SMCRD registry, 16 patients were excluded because of lack of data on inpatient diagnoses, 6927 patients met the inclusion criteria.

Data were collected for patients hospitalized with a diagnosis of influenza (identified using International Classification of Diseases–Tenth Revision codes) at Henry Ford Health System. Data were then deidentified and stored. The study period for influenza patients was from 1st January 2014 to 31st December 2019. A total of 14,174 influenza patients met the inclusion criteria. The EMR queries used for characteristics of the hospital stay and clinical history for the COVID-19 consortium data were the basis of the influenza data queries, so that the two data sources were compatible in definition.

The study patients in both COVID-19 and influenza populations were divided into three groups based on history of atrial arrhythmias (AA): group 1 was the normal sinus rhythm (NSR) group-these patients did not have history of AA and remained in NSR throughout hospitalization; group 2 was the new-onset AA group which did not have a prior history of AA but developed atrial fibrillation or atrial flutter during hospitalization; group 3 patients had a prior history of AA and may have stayed in NSR or experienced AA during hospitalization. The incidence of AA in COVID-19 population was 20.3% with 9% patients having new-onset AA. Within influenza population, 13.1% patients had AA with incidence of new-onset AA at 2.5%.

Hospital records of patients included in the study were reviewed to identify:

1. *Patient characteristics*: Age at first admission, gender, primary race (Black, White, Other).
2. *Patient medical history* Included comorbidities of the patients.
3. *Home medications*: Statins, angiotensin-converting-enzyme inhibitors, angiotensin receptor blockers, beta-blockers, calcium channel blockers, antiplatelets, and antocoagulants.
4. *In-hospital medication use*: Rate controlling agents, rhythm controlling agents, anticoagulants, steroids, anticoagulants, hydroxychloroquine, Remdesivir, tociliumab, lopinavir, and ritonavir.
5. *Dates of service*: First admission date (i.e., date of earliest inpatient encounter for COVID-19 and influenza) and discharge dates.
6. *Patient outcomes*: Included inpatient death (died inpatient versus discharged alive) and new-onset heart failure.

## 3 Echocardiographic Parameters

A total of 115 patients had transthoracic echocardiography performed during the hospital admission. Data on following parameters was reported in our study.

1. *Right ventricular size*: Right ventricular size was stratified into normal, mildly enlarged, moderately enlarged, severely enlarged, and unknown.
2. *Left ventricular size*: Left ventricular size was classified into normal, mildly enlarged, moderately enlarged, severely enlarged, and unknown.
3. *Left ventricular ejection fraction*: Left ventricular ejection fraction was divided into preserved (≥50%), borderline (40–49%), reduced (<40%), and unknown.
4. *Pericardial effusion*: Pericardial effusion was divided into none, small, large, and unknown.
5. *Vavular abnormality*: Valvular abnormalities were divided into none, mild, moderate, severe, and none.
6. *Pulmonary artery systolic pressure*: Pulmonary artery systolic pressure was characterizied into normal (0–40 mmHg), mild elevation (41–50 mmHg), moderate elevation (51–60 mmHg), severe elevation (>60 mmHg), and unknown.

## 4 Statistical Methods

Categorical data were summarized as percentages and fraction of occurrence. Continuous data were summarized as median with interquartile range or means with standard deviations. Variable distributions were compared using Chi-square tests or Fisher's exact tests for categorical data and ANOVA or Kruskal-Wallis tests for continuous data, as appropriate. Generalized linear models were used to estimate odds ratios and risk ratios. A *p*-value of <0.05 was considered significant.

We matched the hospitalized COVID-19 population to a pre-COVID hospitalized viral influenza cohort. Propensity scoring was used serially to generate balanced groups, within the COVID-19 study set, within the influenza study set, and between the COVID-19 and influenza study sets. Logistic regression was used to generate the propensity scores for each stage, using demographics and past medical history variables as predictors. Matching was done using a 0.1sd caliper, without replacement, and with ties broken randomly [12]. Within each study set, logistic regression models were used to first estimate the probability of new-onset AA given that AA were observed (history of AA vs new-onset AA). Since there were few new-onset AA cases in the influenza study set, a 2:1 match was used; 1:1 matching was used for the COVID-19 set. Logistic regression was then used in each study set to model the probability of AA (history/new AA vs none) and cases were matched 1:1. For comparison across the influenza and COIVD-19 cases, we used propensity matching to further align the two study sets. Logistic regression was used to model the probability of a COVID-19 diagnosis as the cause of hospitalization (COVID-19 vs influenza). The studies were matched 1:1. The final study set was achieved with balanced data across AA groups and study sets. Data summaries and analysis were performed with the R programming language (version 4, ggplot2 package) [13].

## Ethics Statements

The study was approved as a retrospective study by institutional review boards at Henry Ford Health System (protocol # 13785) and Trinity Health System (protocol # 2021-009). The need for informed consent was waived for the use of deidentified medical records.

## CRediT authorship contribution statement

**Qasim Jehangir:** Conceptualization, Methodology, Investigation, Writing – original draft, Visualization. **Yi Lee:** Conceptualization, Methodology, Investigation, Writing – original draft, Visualization. **Katie Latack:** Software, Formal analysis, Investigation, Data curation. **Laila Poisson:** Methodology, Software, Formal analysis, Investigation, Writing – original draft. **Dee Dee Wang:** Conceptualization, Resources, Writing – review & editing, Supervision. **Shiyi Song:** Software, Formal analysis, Data curation. **Dinesh R. Apala:** Writing – review & editing. **Kiritkumar Patel:** Conceptualization, Writing – review & editing, Supervision. **Abdul R. Halabi:** Conceptualization, Writing – review & editing, Supervision. **Geetha Krishnamoorthy:** Resources, Writing – review & editing, Supervision. **Anupam A. Sule:** Conceptualization, Methodology, Resources, Writing – review & editing, Supervision, Project administration.

## Declaration of Competing Interest

The authors declare that they have no known competing financial interests or personal relationships that could have appeared to influence the work reported in this paper.

## Data Availability

Data of Atrial Arrhythmias in Hospitalized COVID-19 and Influenza Patients (Original data) (DIB).
